# AI interventions as hypothesis cues: rethinking overreliance in human-AI decision-making

**DOI:** 10.3389/fpsyg.2026.1967356

**Published:** 2026-10-06

**Authors:** Mai Phuong Pham, David A. Illingworth-Vera, Rickey P. Thomas

**Affiliations:** 1 School of Psychological and Brain Sciences, Georgia Institute of Technology, Atlanta, GA, United States; 2 Department of Psychology, California State University, Long Beach, Long Beach, CA, United States

**Keywords:** decision making, explainable AI, human-AI interaction, hypothesis generation, memory, overreliance

## 1 Introduction

The proliferation of human-AI systems has intensified concerns about the misuse of AI technologies (Parasuraman and Riley, 1997; Amershi et al., 2019). A growing literature has examined cases of misuse with a keen interest in miscalibrated use, specifically overreliance. Currently, numerous interventional methods have emerged to mitigate the improper integration of AI recommendations in decision-making processes. These interventions have had mixed results, as some appear to exacerbate the issue by implying AI technologies are better calibrated to an operator's task than they truly are. We argue that such results may be a consequence of hyper-fixation on behavioral outcomes and tool escalation, while failing to advance theoretical accounts for how AI outputs are integrated into cognitive processes.

Here, we use explainable AI (XAI) as an example intervention for illustrating the influence of AI interventions on overreliance. Specifically, we propose that AI interventions prematurely cue and privilege one interpretation of the decision space before competing alternatives have been sufficiently activated. In this view, what is typically characterized as blind acceptance of an AI recommendation may instead reflect an inadequately populated comparison set, in which plausible alternatives were never retrieved, maintained, or activated strongly enough to compete with the AI-supported interpretation. Thus, the acceptance of AI recommendations may reflect a constrained decision-making process rather than poorly calibrated trust.

Figure 1 illustrates the proposed XAI-as-hypothesis-cue framework, in which AI recommendations and explanations influence trust and reliance by shaping the hypotheses available for comparison. Task cues and AI outputs activate candidate hypotheses, while explanations can either strengthen the AI-supported interpretation (focal hypothesis) or activate plausible alternatives. These effects depend on cognitive and task constraints. When multiple alternatives remain active, operators are more likely to engage in diagnostic search and exhibit calibrated reliance. When the AI-supported hypothesis becomes dominant, search is more likely to become confirmatory, increasing the risk of overreliance.

**Figure 1:**
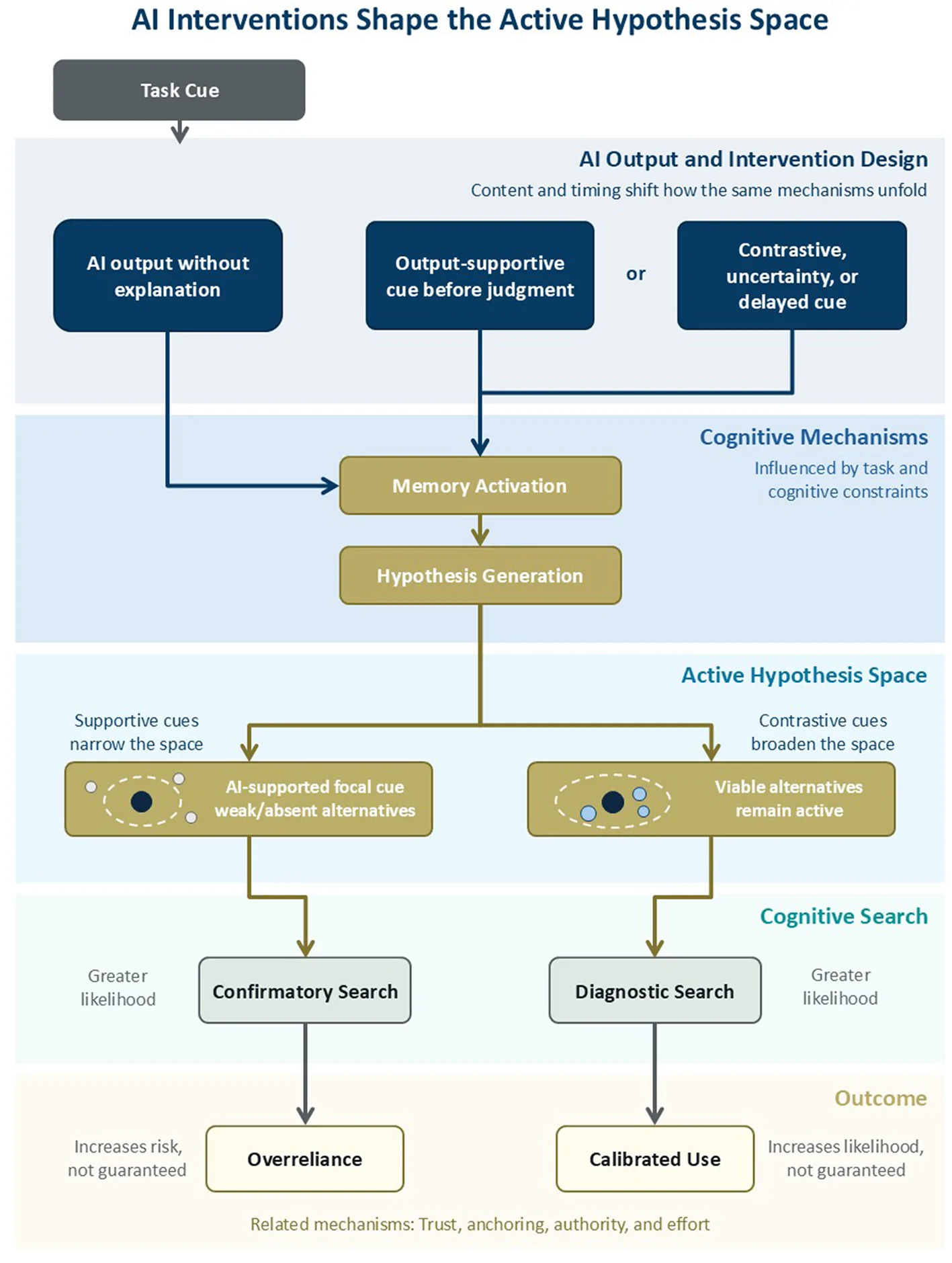
The hypothesis-generation framework of XAI overreliance. Task cues, model outputs, and explanations activate candidate hypotheses. When viable alternative hypotheses are active, diagnostic search and calibrated reliance become more likely. When the AI-supported hypothesis becomes focal and alternatives are weak or absent, confirmatory search becomes more likely, increasing the risk of overreliance. The arrows indicate proposed influences whose effects depend on cognitive and task conditions; neither search patterns nor reliance outcomes are inevitable.

## 2 Overreliance in the context of XAI

Many accounts frame overreliance as a problem of excessive trust, insufficient monitoring, or opacity in AI output (Endsley and Kiris, 1995; Hoff and Bashir, 2015). XAI addresses this problem by making model recommendations more interpretable, with the expectation that operators can better evaluate why an output was produced (Lee and Moray, 1992; Ribeiro et al., 2016). This approach can be effective in relatively simple or well-structured tasks, especially when AI recommendations are accurate and explanations clarify task-relevant cues (Bansal et al., 2021). However, calibrating reliance becomes more difficult in complex or uncertain decision environments, where model outputs may reflect epistemic uncertainty, aleatory uncertainty, or hallucinations (Parmar et al., 2021; Ashktorab et al., 2025).

Consistent with this limitation, richer explanations can increase trust and overreliance even when incorrect (Bussone et al., 2015; Bansal et al., 2021). By compressing task cues and model reasoning into digestible chunks, explanations may encourage model-consistent search (Payne et al., 1993; Pirolli and Card, 1999). Under time pressure or cognitive load, this can anchor operators to the model's characterization of the problem, impair error detection, and increase acceptance without improving complementary performance (Poursabzi-Sangdeh et al., 2021; Bansal et al., 2021).

Process-level interventions, such as cognitive forcing, provide further evidence that the sequence of reasoning matters. Requiring operators to form an independent judgment before viewing AI advice can reduce overreliance (Buçinca et al., 2021). Yet, such interventions are often interpreted as improving post-hoc evaluation of AI advice through greater effort or more cautious trust calibration. This interpretation leaves open another possibility that delaying AI input may preserve the opportunity to retrieve and consider competing hypotheses before an AI-supported interpretation becomes focal.

## 3 Why hypothesis generation matters

From a memory-theoretic perspective, judgment and decision-making behaviors are grounded in the hypotheses retrieved from and maintained within memory systems. Available task information acts as a set of retrieval cues that activate candidate hypotheses (Thomas et al., 2008). These processes are constrained by information search costs, restricted by cognitive load (Sprenger et al., 2011), working memory capacity (Dougherty and Hunter, 2003), time pressure (Dougherty and Hunter, 2003), and other resources (Böckenholt and Weber, 1993; Weber et al., 1993; Thomas et al., 2014; Illingworth and Thomas, 2022; Payne et al., 1993; Pirolli and Card, 1999). Specifically, under time pressure or cognitive load, people can maintain only a limited set of active alternatives (Dougherty and Hunter, 2003). Early cues shape which hypotheses are retrieved, maintained, used to form confidence, and used to guide further search (Dougherty et al., 2010; Illingworth and Thomas, 2022; Lange et al., 2013).

Research in diagnostic reasoning suggests that the precursors of overreliance emerge well before belief evaluation, at the point of hypothesis generation. Work on the temporal dynamics of hypothesis generation has shown that early cues, data serial order, and the first hypotheses generated can exert a primacy-like influence on subsequent diagnostic reasoning (Böckenholt and Weber, 1993; Lange et al., 2012). Initial hypotheses shape which evidence is sought, how subsequent information is interpreted, and whether the decision-maker ultimately arrives at the correct diagnosis (Böckenholt and Weber, 1993; Illingworth and Thomas, 2022). Related work on counterfactual forecasting suggests that better decisions may require increasing the number and diversity of generated hypotheses, not only improving explanations (Illingworth et al., 2023). Model-blindness findings similarly suggest that cueing effects depend on whether feedback and explanations review model limitations, allowing operators to reassess the diagnostic value of model outputs and reducing the likelihood that model recommendations dominate the hypothesis space (Parmar et al., 2023). Together, these findings motivate the idea that the set of alternatives available for comparison may be determinative of whether the subsequent AI output is appropriately evaluated.

The hypothesis-cue account overlaps with, but is not reducible to, neighboring concepts of AI overreliance. Automation-bias and authority accounts emphasize excessive deference to automated recommendations (Skitka et al., 1999; Hoff and Bashir, 2015; Lee and Moray, 1992); anchoring invokes the heuristic mechanism of insufficient adjustment from the initially presented conclusions (Epley and Gilovich, 2006); effort-reduction accounts emphasize the switch to less demanding strategies (Payne et al., 1993; Buçinca et al., 2021); and confirmation bias accounts emphasize the preferential testing of an already focal belief (Klayman and Ha, 1987). Our distinct claim concerns a change in the alternatives available for evaluation at an earlier stage where AI output can alter the number, diversity, accessibility, and activation strength of the hypotheses in the operator's active comparison set (Dougherty and Hunter, 2003; Thomas et al., 2008; Illingworth and Thomas, 2022). Accordingly, two operators may report equal trust in an AI system, but rely on it differently because different alternatives are available for comparison. Thus, the framework predicts that the timing and content of explanations will affect alternative generation and information search even after perceived trust, authority, and effort are controlled.

## 4 AI explanations as hypothesis cues

The findings reviewed in the previous section point to a broader account of overreliance in which XAI interventions shape not only how operators evaluate a recommendation but also how recommendations are integrated into reasoning processes and influence the construction of the alternative hypothesis set. We next consider how this process varies with the presence, content, and timing of an AI explanation.

### 4.1 AI output without explanation

Our claim is not that AI interventions inevitably narrow the hypothesis space. Even an AI output without an explanation attached can cue one hypothesis by making it focal (Skitka et al., 1999; Epley and Gilovich, 2006). Because a stripped-down recommendation often provides little supporting structure, its effect will likely depend on perceived model competence and whether alternative hypotheses are already active (Hoff and Bashir, 2015). Even so, early presentation can give an AI-prompted hypothesis temporal and retrieval advantages that shape the interpretation of later evidence (Böckenholt and Weber, 1993).

### 4.2 AI output with a supportive explanation

Adding a supportive explanation can do more than increase credibility. By supplying model-consistent cues and compressing the model's reasoning into a fluent rationale, the explanation may elaborate the recommended hypothesis, increase its accessibility, and reinforce model-consistent search or undermine the perceived usefulness of disconfirmatory search (Bansal et al., 2019; Dougherty et al., 2010; Payne et al., 1993; Pirolli and Card, 1999). Subsequent reasoning may appear careful even though it occurs within an impoverished comparison set. That is, operators may inspect evidence and articulate a coherent rationale without retrieving viable alternatives. The explanation can then guide confirmatory rather than discriminating search (Klayman and Ha, 1987; Illingworth and Thomas, 2022), increasing reliance without improving complementary performance (Poursabzi-Sangdeh et al., 2021; Bansal et al., 2021).

### 4.3 AI output with a contrastive or uncertainty-focused explanation

An explanation that identifies plausible alternatives, specifies why the recommendation was favored over them, or indicates what evidence would discriminate among them can broaden the active comparison set (Miller, 2019; Illingworth et al., 2023). These explanations should have qualitatively different effects because expanding the hypothesis space can support calibration and orient information search toward evidence to discriminate among alternatives. However, explanations that display uncertainty alone may calibrate perceived reliability (e.g., through a confidence percentage display) without helping operators determine what alternative to consider. Therefore, the effectiveness of such interventions may depend on how well the uncertainty signal is calibrated to the model's actual reliability (Lemus et al., 2023). Accordingly, uncertainty should be linked to model error, misspecifications, missing information, and competing hypotheses if it is to influence hypothesis generation and evaluation rather than trust alone (Zhang et al., 2020; Parmar and Thomas, 2020; Parmar et al., 2023).

### 4.4 Intervention before versus after independent judgment

Sequencing should determine whether the intervention helps construct the initial hypothesis set or is evaluated against alternatives already generated. When presented before independent judgment, a supportive intervention may give one hypothesis both primacy and greater elaboration. Presented after an initial judgment or explicit alternative-generation step, the same explanation is more likely to encounter an already populated comparison set. Cognitive forcing may therefore reduce overreliance not simply by lowering trust or increasing effort, but by preserving an opportunity to retrieve alternatives before AI guidance becomes dominant (Buçinca et al., 2021). Thus, the framework predicts an interaction where early supportive explanations should narrow consideration of alternative hypotheses most strongly, whereas contrastive explanations and delayed presentation should promote alternative hypothesis generation and diagnostic comparisons.

## 5 Discussion

The practical implication of the proposed framework is straightforward in that operators should form an independent judgment and generate at least one plausible alternative before viewing an AI recommendation or its explanation. Operators should be prompted to ask what evidence would distinguish the AI-supported hypothesis from its strongest competitor and what observation would indicate that the AI is in error. When feasible, recording an initial assessment before consulting AI can separate hypothesis generation from AI evaluation. The purpose is not to discourage trust, but to ensure that reliance follows comparison among plausible alternatives rather than the fluency of a single AI-supported interpretation (Klayman and Ha, 1987; Illingworth and Thomas, 2022).

For designers, the framework shifts the focus upstream to hypothesis generation. Systems should delay recommendations when early outputs are likely to dominate, prompt operators to generate alternatives, present explanations that compare hypotheses, and identify potentially discriminating evidence, rather than merely justify the model's answer. Interfaces can highlight missing evidence and diagnostic task information to discriminate among competing hypotheses, as well as alert operators when they fall prey to confirmation bias (when their search is focused on a single option). In this role, AI facilitates the mapping of the decision space before promoting a preferred conclusion. Moreover, probabilistic support may externalize uncertainty, although its value will likely depend on whether it expands what operators attend to and consider rather than simply making recommendations appear more precise and trustworthy.

For researchers, we believe that they should consider that reliance, trust, and accuracy are insufficient outcome measures. Studies should also address the number and diversity of hypotheses generated, their rank or relative activation, the information operators select, and whether search is confirmatory or diagnostic (Dougherty and Hunter, 2003; Thomas et al., 2008; Illingworth and Thomas, 2022). The presence of the recommendation, the type of explanation, and the timing should be manipulated separately to determine whether an intervention changes trust, the set of active hypotheses, or both. A key prediction is that reliance can change without a corresponding change in reported trust when AI output alters the alternatives available for comparison.

Thus, the central design challenge is not only to make AI recommendations comprehensible, but to prevent a single AI-consistent interpretation from becoming the only option seriously considered by the operators. Explanations should be most useful when they support comparison, review model limitations, and preserve access to viable alternatives before commitment.
